# A Web Tool for Consensus Gene Regulatory Network Construction

**DOI:** 10.3389/fgene.2021.745827

**Published:** 2021-11-24

**Authors:** Chiranjib Sarkar, Rajender Parsad, Dwijesh C. Mishra, Anil Rai

**Affiliations:** ^1^ ICAR-Indian Agricultural Research Institute, New Delhi, India; ^2^ ICAR-Indian Agricultural Statistics Research Institute, New Delhi, India

**Keywords:** web tool, PHP, fisher’s weighted method, consensus approach, gene regulatory network

## Abstract

Gene regulatory network (GRN) construction involves various steps of complex computational steps. This step-by-step procedure requires prior knowledge of programming languages such as R. Development of a web tool may reduce this complexity in the analysis steps which can be easy accessible for the user. In this study, a web tool for constructing consensus GRN by combining the outcomes obtained from four methods, namely, correlation, principal component regression, partial least square, and ridge regression, has been developed. We have designed the web tool with an interactive and user-friendly web page using the php programming language. We have used R script for the analysis steps which run in the background of the user interface. Users can upload gene expression data for constructing consensus GRN. The output obtained from analysis will be available in downloadable form in the result window of the web tool.

## 1 Introduction

Gene regulatory network (GRN) construction is important for understanding complex biological processes. GRNs are represented as the nodes connected with edges where the nodes indicate the genes and each edge indicates the strength of the relationship between the genes. GRNs are constructed from high-dimensional gene expression data containing thousands of genes with expression values at different conditions or experiments. It is a computationally challenging task for analyzing high-dimensional gene expression data in a stepwise workflow. Constructing a GRN from gene expression data involves various steps of data analysis. The steps involved in GRN construction required use of computational techniques. Prior knowledge of the programming language is required for analyzing gene expression data as well as network construction. There are different statistical methods proposed for inferring GRN from high-dimensional expression data, and these methods are implemented using different R packages available in the CRAN depository. Some of the proposed statistical methods are implemented with online web tools. R packages like “BNArray” (Chen et al., 2006), “minet” (Meyer et al., 2008), “dna” (Gill et al., 2014), and “ENA” (Allen, 2014) are implemented based on the Bayesian network, mutual information, differential network analysis methods, and ensemble network aggregation, respectively. Instead of executing a script for each step of GRN construction, web tool development may provide easy accessibility to the user. There are some web tools developed for GRN construction like MIDER (Villaverde et al., 2014), NetworkAnalyst (Zhou et al., 2019), CoExpNetViz (Tzfadia et al., 2016), and GeNeCK (Zhang et al., 2019). For easy accessibility and to provide a more user-friendly procedure, we have introduced a web tool for constructing consensus GRN. It allows users to provide their own gene expression data to get the significant edges and nodes of the GRN. In our web tool, we have used Fisher’s weighted method for combining the output of GRN obtained from correlation, principal component regression (PCR), partial least square (PLS), and ridge regression (Sarkar et al., 2020). The data analysis part of computing the edge score from correlation, PCR, PLS, and ridge regression has been written in R programming language. The web pages were designed using the HTML and php languages with a user-friendly interface. Users can provide the input file in Microsoft Excel format, and the output of significant edges in each step will also be provided in Excel format.

## 2 Program Description and Methods

**FIGURE 1:**
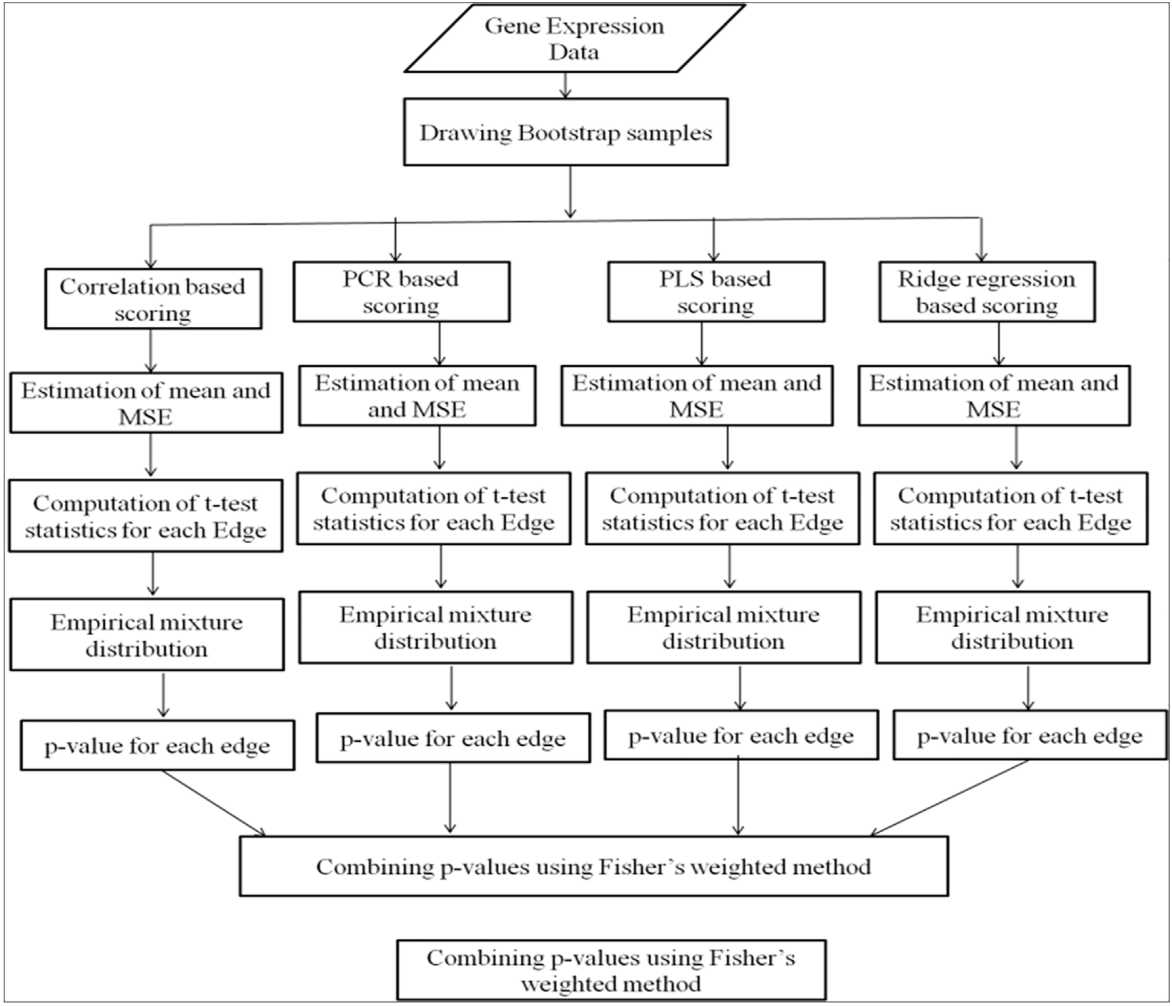
Workflow of web tool for consensus GRN construction.

Our developed web tool mainly follows three steps—data uploading, data analysis, and combining the outputs of four methods. The input data of gene expressions can be provided in comma separated value (.csv) file format or in Microsoft excel format containing the list of genes in rows and the conditions or various experiments in columns. The user-uploaded input data are renamed with the date and time of data uploading to avoid repetition in the uploaded file name. The edge scores are computed using four methods, *i.e.*, correlation, PCR, PLS, and ridge regression methods. Probability values are computed for edges from the mixture distribution of edge scores obtained from each method. The probability values are combined using Fisher’s weighted method (Figure 1). Different steps of analysis are done using R programming. Few R packages like “dna” and “fdrtool” are used in writing the R script for the analysis. The outputs of the analysis are available in downloadable format in the result tab. Each output file contains the names of the interacting genes and the connectivity score.

### 2.1 Design of the Web Tool

**FIGURE 2:**
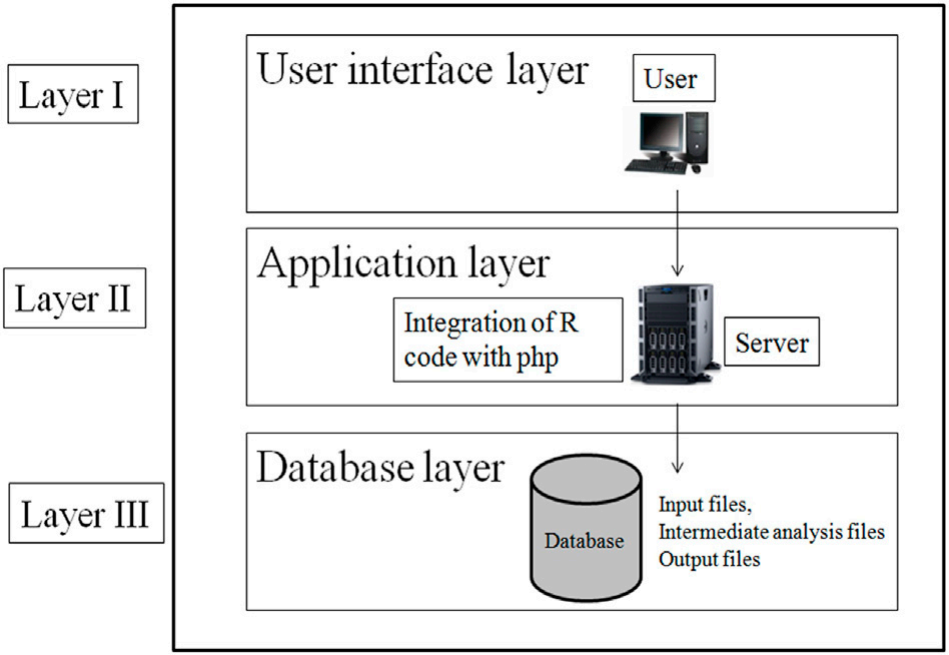
Three layer architecture of web tool for consensus GRN construction.

The web tool has been designed using standard three-layer web architecture (Figure 2). The three layers of web architecture are:

• Layer I—user interface layer (UIL)
• Layer II—application layer (APL)
• Layer III—database layer (DBL)

#### 2.1.1 User interface layer

The UIL for the web tool was developed using HTML (Hyper Text Markup Language), CSS, and JavaScript. The UIL consists of forms to interact with users. In UIL, users can upload the gene expression dataset in excel format and download the result file.

#### 2.1.2 Application Layer

The APL of the web tool has been designed using php and R code. The R script for constructing GRN has been integrated with php for analysis of gene expression data. The R script is executed in the background of the web tool which is not visible to the user.

#### 2.1.3 Database Layer

The DBL has been designed as server side file storage. This layer stores the user-provided input file, the intermediate files generated in R script execution, and the final result file. Intermediate files are like files containing a pairwise scoring matrix from four individual methods: file containing *p*-value, fdr value, and *F*_*w*_ score.

The php scripts and R scripts are given in the Supplementary File.

### 2.2 Data Analysis

The expression values of genes in the input data file are considered for computing the connectivity score of each pair of genes using correlation, PCR, PLS, and ridge regression. Bootstrap samples are drawn from the input dataset. The “Sample” function has been used to draw bootstrap samples in R script. For each bootstrap sample, the connectivity score is computed using the four methods. The probability values of pair of genes are computed to measure the statistical significance of the connectivity of gene pair. The probabilities of gene pairs are obtained from the mixture distribution of the connectivity scores of all possible pairs of genes (Efron, 2004).

The correlation-based connectivity score (Gill et al., 2010) is:

$$S_{ik}=\frac{x_{i}^{T}x_{k}}{\sqrt{\left(x_{i}^{T}x_{i}\right)\left(x_{k}^{T}x_{k}\right)}}$$ (1)

where *x*_*j*_ and *x*_*k*_ are the standardized expression values of the i^th^ and k^th^ genes, respectively, and $S_{ik}$ is the connectivity score between the *i* ^*th*^ and *k* ^*th*^ genes.

The PCR-based connectivity score (Pihur et al., 2008) is:

$${\left[s_{g1},...,s_{g,g-1},s_{g,g+1},...s_{gp}\right]}^{T}=V{\hat{\text{β}}}_{g}$$ (2)

where $s_{gp}$ is the connectivity score between the *g*th and *p*th genes and V is the matrix of eigen vectors computed from gene expression values.

### The PLS-Based Connectivity Scoring Is

$${\hat{s}}_{ik}=\frac{\sum_{l=1}^{v}{\hat{\beta }}_{il}c_{ik}^{\left(l\right)}+\sum_{l=1}^{v}{\hat{\beta }}_{kl}c_{ik}^{\left(l\right)}}{2}$$ (3)

where

$${\hat{\beta }}_{il}={\left(t_{i}^{{\left(l\right)}^{T}}t_{i}^{\left(l\right)}\right)}^{-1}t_{i}^{{\left(l\right)}^{T}}x_{i}$$

$$t_{i}^{\left(l\right)}=\sum_{k\neq i}^{p}c_{ik}^{\left(l\right)}X_{k}^{\left(l\right)}$$

$$c_{ik}^{\left(l\right)}=\frac{X^{{\left(l\right)}^{T}}x_{i}}{\sqrt{x_{i}^{T}X^{\left(l\right)}X^{{\left(l\right)}^{T}}x_{i}}}$$

The ridge regression-based connectivity score (Gill et al., 2010) is:

$${\left[s_{g,1},...,s_{g,g-1},s_{g,g+1},...,s_{g,p}\right]}^{T}={\left({\tilde{X}}_{g}^{T}{\tilde{X}}_{g}+\lambda I\right)}^{-1}{\tilde{X}}_{g}x_{g}$$ (4)

where *s*_*gp*_ is the connectivity score between the *g*th and *p*th genes.

The computation of the connectivity scores was implemented using the “dna” R package.

The mean and standard error (SE) are calculated as (Sarkar et al., 2020):

$${\bar{s}}_{ik}=\frac{\sum_{i\neq k}^{n}\sum_{j=1}^{B}s_{ik_{j}}}{B}$$ (5)

$$\begin{array}{l} Se=\frac{1}{\sqrt{B-1}}\sqrt{\sum_{i\neq k}^{n}\sum_{j=1}^{B}{\left(s_{ik_{j}}-{\bar{s}}_{ik}\right)}^{2}} \end{array}$$ (6)

where B is the number of Bootstrap samples.

The computed *t*-test statistic is as follows:

$$t=\frac{{\bar{s}}_{ik}}{Se}$$ (7)

For the correlation-based scoring method, the *t*-test statistic is computed as follows:

$$t=\frac{{\bar{s}}_{ik}\sqrt{n-2}}{\sqrt{1-{\bar{s}}_{ik}^{2}}}$$ (8)

The t-statistic values are used for mixture distribution estimation using the “fdrtool” R package (Klaus and Strimmer, 2015).

The *p*-values are combined using Fisher’s weighted method (Hedges and Olkin, 2014) following the steps as given in Sarkar et al. (2020):

$$F_{w}=-2\ln\left(p_{1}\times p_{2}\times p_{3}\times p_{4}\right)$$ (9)

### 2.3 Implementation

**FIGURE 3:**
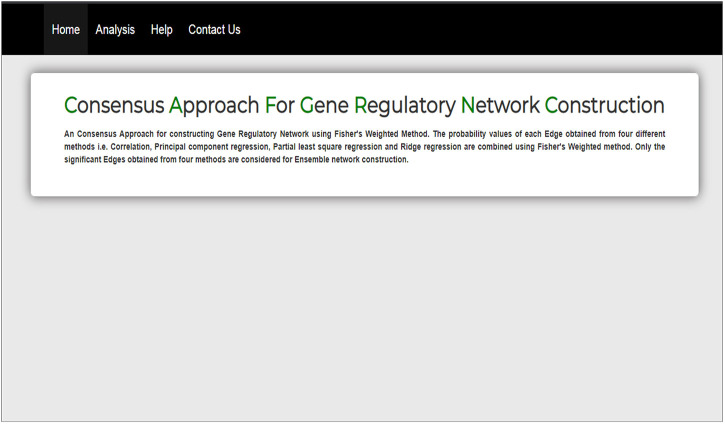
Interface of homepage of web tool.

**FIGURE 4:**
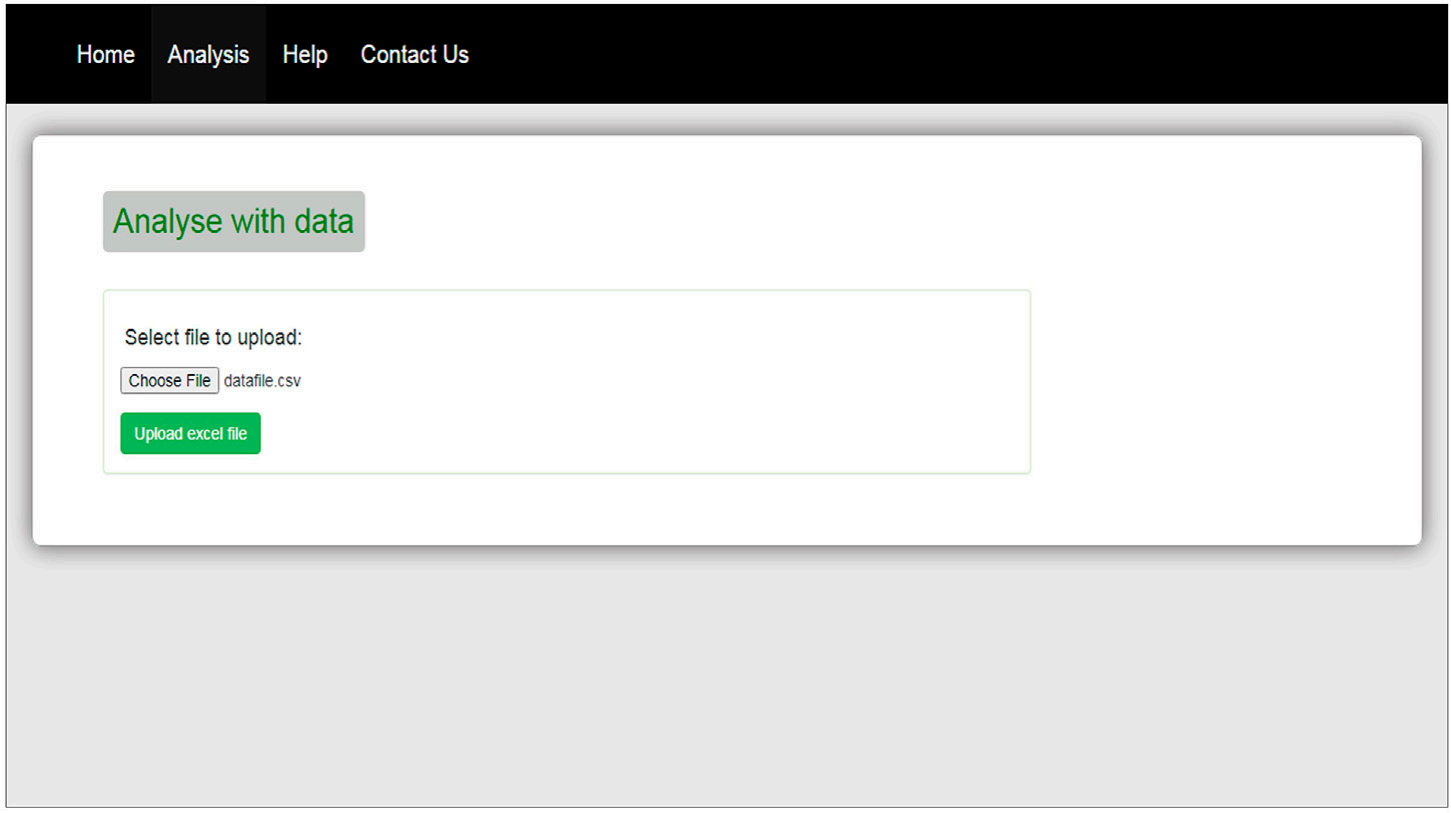
The upload option in analysis tab of web tool.

**FIGURE 5:**
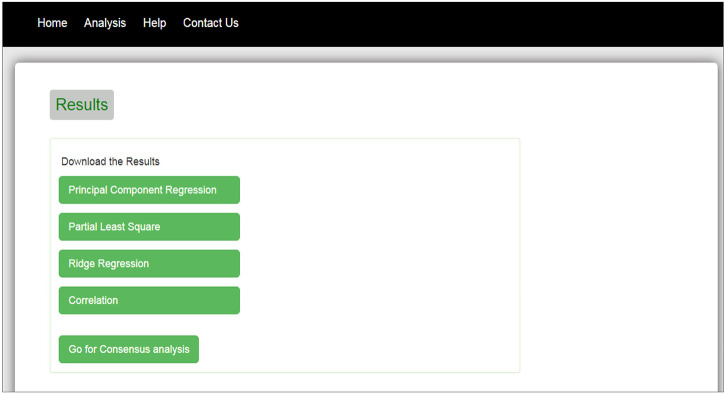
Download tab of results obtained from four methods.

**FIGURE 6:**
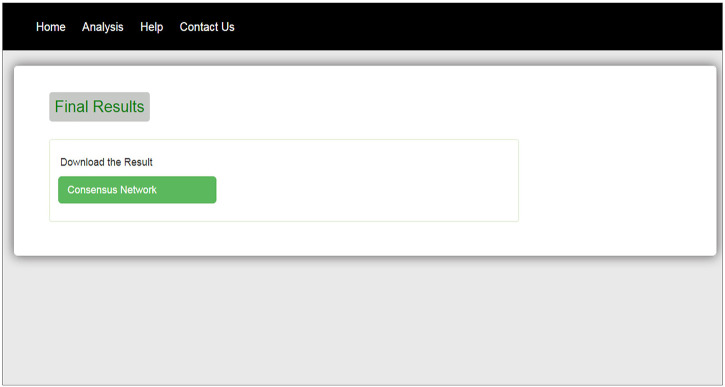
Download tab for final result.

The interface of our web tool has four tabs “Home,” “Analysis,” “Help,” and “Contact Us” (Figure 3). The “Analysis” tab has an option to upload gene expression data (Figure 4). The input file format of gene expression values should be in comma-separated values (csv) or Excel with genes in rows and conditions in columns. The output files are available in Excel format in the download tab of each method (Figure 5). The output file consists of edges, connectivity scores of edges, fdr values, and *p*-values of each edge. The *p*-values of edges computed from the four methods are combined using Fisher’s weighted method, and the combined result is available in downloadable format (Figure 6). The final output file contains the lists of the significant edges with F-score. The final result file contains the edges for consensus GRN.

## Discussion

In this study, a web tool named “Consensus Approach for Gene Regulatory Network Construction” for GRN construction has been developed which provides the network file containing the edge scores of significant interactions of gene pairs. The output file can be visualized using network visualization tools like Cytoscape. In our web tool, we provide the output file containing all the score and statistic values obtained from four individual methods which can also be visualized in Cytoscape. The web tool is easy to use in that it does not require any prior knowledge of R programming and computational steps. It will be very easy for users to construct GRN from gene expression data.

## Data Availability

The original contributions presented in the study are included in the article/Supplementary Material, further inquiries can be directed to the corresponding author.
