# Supplementary material for: A Web Tool for Consensus Gene Regulatory Network Construction
Source: Front Genet. 2021 Nov 24;12:745827. doi: 10.3389/fgene.2021.745827 (PMC8652126; doi:10.3389/fgene.2021.745827)
Supplement: Supplementary file 2 [file Table1.docx]

Table S1. Gene expression datasets and their accession numbers available in NCBI Database

| Accession No. | No. of samples | Title |
| --- | --- | --- |
| GDS3441 | 8 | Rice leaves response to blast fungus infection: time course |
| GSE61952 | 6 | Expression data from rice lesion mimic mutant spotted leaf 5 (spl5) |
| GSE41798 | 18 | Transcriptome study of rice early response to rice blast fungus |
| GSE30941 | 15 | Rice gene global expression analysis upon inoculation with different Magnaporthe isolates |
